# An Analysis of the 50 Most-Cited "Uveitis" Articles Published Between 2010-2020 From a Bibliographic and Altmetric Perspective

**DOI:** 10.7759/cureus.29930

**Published:** 2022-10-04

**Authors:** Hidayet Sener, Cem Evereklioglu, Fatih Horozoglu

**Affiliations:** 1 Department of Ophthalmology, Erciyes University School of Medicine, Kayseri, TUR

**Keywords:** immediacy index, eigenfactor, impact, altmetric, ocular inflammation, uveitis

## Abstract

Purpose

We aimed to evaluate the 50 most-cited articles on uveitis according to their Altmetric Attention Score (AAS) and additional metrics.

Methods

The Web of Science (WoS) core collection database was used in this study. The article and journal metrics and characteristics were examined. In addition, the effect of article and journal metrics on the AAS was examined with multivariate adaptive regression splines (MARS).

Results

The number of citations of the evaluated articles ranged from 670 to 90, and AASs ranged from 633 to 0. According to the MARS model, the importance scores of the predictors were as follows: article influence score (100%), immediacy index (77.74%), number of years since publication (57.79%), times cited in WoS (32.69%). We found that the trend of articles on uveitis was related to the "treatment category", namely, adalimumab. Second, the popular topic was uveitis caused by viruses.

Conclusions

We found that citation-based metrics and year of publication contributed to AAS. AAS appears to be inadequate in assessing the quality of articles. However, due to the electronic transformation of the publishing industry, it seems inevitable that altmetrics will become an additional supportive metric.

## Introduction

Uveitis refers to inflammation of the uveal tract, which can cause loss of vision [1]. The incidence of uveitis peaks at working ages [2]. Blindness caused by uveitis is treatable or potentially preventable. Not all types of uveitis have the same effect on vision, and uveitis should be classified and treated according to the underlying etiology. Systemic immunomodulatory agents and conventional immunosuppressive agents are available for the treatment of non-infectious uveitis (NIU). In recent years, the introduction of biological agents has led to important developments in the treatment of uveitis. Biological agents are promising drugs designed to be effective based on the molecular understanding of disease pathogenesis [3]. Infectious uveitis (IU) is one of the most common and visually devastating causes of uveitis worldwide [4]. IUs can mimic NIUs and their early diagnosis and treatment are important.

The main purpose of authors is to convey scientific information to a wide audience. The impact of the articles is evaluated by various metric measurements. The most important of these is the number of times cited. Another important point is the metrics of the journal publishing the article. The most well-known of these is the impact factor (IF), which is calculated by examining the citations of the articles in scientific journals and showing the impact of the journals in their fields [5].

Citations are of course important in calculating journal metrics. However, nowadays, due to the development of the internet and social media, scientific articles are not only cited from scientific journals but also from many sources. Considering that it would be incomplete to evaluate the popularity of a scientific publication by the number of scientific citations alone, altmetrics were created that include citations in channels such as social media, Twitter, blog posts, and news sites. Altmetrics compile the impact of the article on the internet and social media from dozens of different sources and scores its popularity. Altmetrics also measure where and by whom the article is cited, such as language, country, and region [6].

In this study, we aimed to evaluate the 50 most-cited articles on uveitis according to their Altmetric Attention Score (AAS) and additional metrics. The journals in which the articles were published, categories of the journals, the types of articles, and the topic of the article were examined. In addition, we aimed to show the effect of article and journal metrics on the AAS.

## Materials and methods

The Web of Science (WoS) core collection database (www.webofknowledge.com) was used in this study. In the advanced search section, the term "Uveitis" was searched in the title, abstract, and keywords. The 50 most-cited articles were included in the study (Access date: February 17, 2022). The time filter was applied between January 01, 2010, and December 31, 2020. No other filters were applied. All data obtained were collected on the date of access. The list of articles obtained is presented in the appendices. Non-relevant articles and animal studies were excluded. Relevant metrics of all articles were obtained. We focused on articles whose primary priority was uveitis.

Titles, first authors, all authors, year of publication, number of years since publication (NYsP), number of citations, Altmetric Attention Score (AAS) and average citations per year (ACpY), journal IF, journal 5-year IF, Q category, journal citation indicator (JCI), Eigenfactor score (EF), article influence score (AIS), immediacy index (II) and h-index were recorded and examined.

The AASs of the articles were obtained from the bookmark "Altmetric it!" (www.altmetric.com). The h-index of journals was obtained from scientific journal rankings (www.scimagojr.com). All other metrics were obtained from the WoS and journal citation report 2020 (www.jcr.clarivate.com). All statistical analyses were performed using Minitab 17 Statistical Software (Minitab, Inc., State College, PA) and Salford Predictive Modeller software v. 8 (Minitab, Inc.). Kolmogorov-Smirnov normality test was performed with the data set. The data were non-normally distributed and medians (25-75% quartiles) were used. Metric data were evaluated with Spearman’s rho correlation analysis. In evaluating the relationship between the AAS and additional metrics, multivariate adaptive regression splines (MARS), which can establish both a linear and non-linear model, were used while creating the regression model. The eight predictors included in the model were: times cited in WoS, 5-year IF, normalized EF, AIS, II, NYsP, and h-index.

## Results

**Figure 1:**
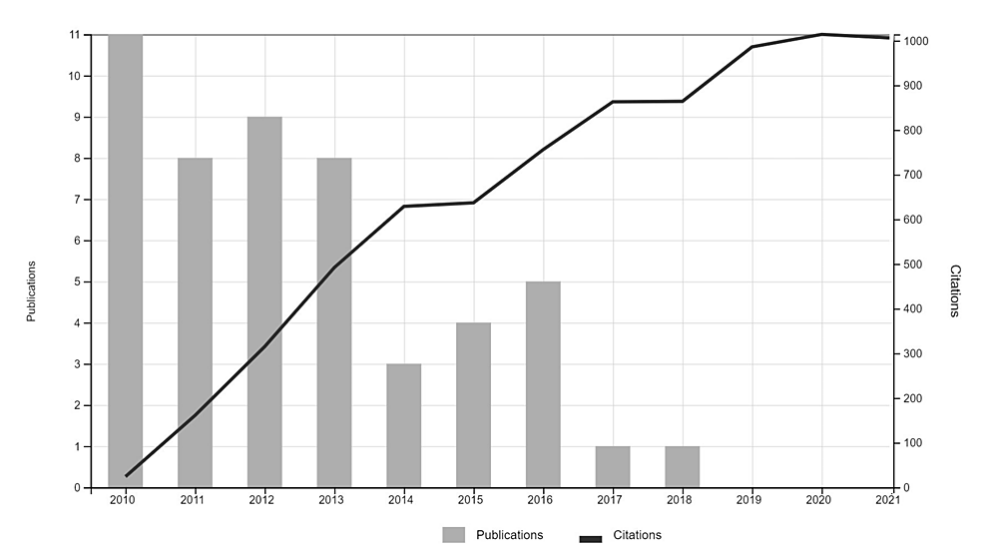
Cumulative display of citations by year and publication years of the articles included in the study

**Figure 2:**
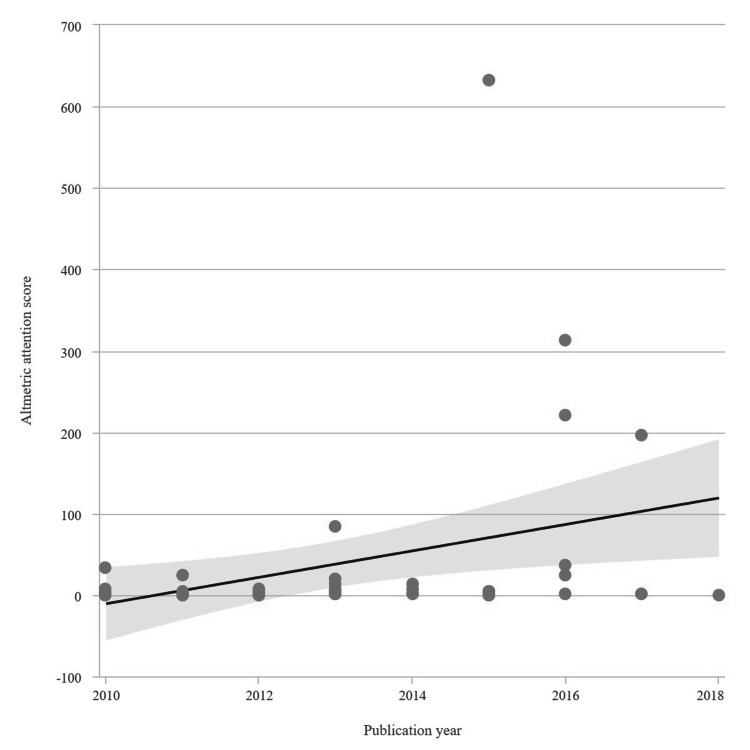
Linear regression graph showing the relationship between publication year and Altmetric Attention Score of the articles included in the study

The number of citations of the evaluated articles ranged from 670 to 90, and AASs ranged from 633 to 0. Detailed metrics and characteristics of the first 50 uveitis articles are summarized in Table 1. The top 50 articles were cited 7897 times. The relationship between the year of publication of the articles and the annual cumulative citation is presented in Figure 1. The scatter plot of AAS by years is presented in Figure 2.

**Table 1:** Metrics and characteristics of the 50 most-cited articles Median (25%-75% interquartile range), Mean±Standard deviation, NA: not applicable, ACpY: average citation per year, NYsP: number of years since publication, WoS: Web of Science

	N	Times cited of WoS	Altmetric Attention Score	Publication year	NYsP	ACpY of WoS
All articles	50	123 (103.0-166.75)	3 (1.0-9.25)	2012 (2011.0-2015.0)	10 (7.0-11.0)	13.9 (10.7-18.2)
Non-infectious uveitis (NIU)	38	121.5 (102.0-173.25)	3 (1.0-6.5)	2012 (2010.0-2014.25)	10 (7.75-12.0)	13.6 (10.4-18.8)
Infectious uveitis (IU)	5	113 (97.5-208.5)	1 (0.5-427.0)	2015 (2013.0-2015.5)	7 (6.5-9)	17.1 (11.6-29.7)
NIU + IU	7	136 (111.0-156.0)	7 (3.0-20.0)	2013 (2012.0-2014.0)	9 (8.0-10.0)	13.8 (12.2-17.3)
Study type						
Systematic reviews and meta-analysis	1	140	84	2013	9	15.5
Reviews	9	113 (105.0-169.5)	1 (0.0-5.5)	2014 (2011.0-2016.0)	8 (6.0-11.0)	16.3 (12.3-22.6)
Original research	39	122 (103.0-166.0)	3 (1.0-14.0)	2012 (2011.0-2014.0)	10 (8.0-11.0)	13.5 (10.4-17.1)
Cohort study	13	109 (95.0-146.0)	1 (1.0-5.0)	2012 (2010.0-2013.0)	10 (9.0-12.0)	12.2 (10.0-14.4)
Randomized controlled trial	9	202 (143.0-349.0)	24 (5.0-116.5)	2013 (2010.5-2016.0)	9 (6.0-11.5)	34.2 (15.1-43.0)
Case report	3	103 (NA)	221 (NA)	2015 (NA)	7 (NA)	17.1 (NA)
Case series	10	118 (107.0-140.0)	1 (0.75-3.0)	2012 (2010.0-2012.25)	10 (9.75-12.0)	11.3 (9.6-14.9)
Cross-sectional study	4	120.5 (102.0-150.25)	0.5 (0.0-10.0)	2011.5 (2011.0-2013.5)	10.5 (8.5-11.0)	13.4 (10.0-14.1)
Conference paper	1	267	8	2014	8	33.3
Study topic						
Full review	4	112.5 (107.5-163.25)	1 (0.25-5.5)	2013.5 (2011.5-2016.25)	8.5 (5.75-10.25)	15.2 (11.5-20.8)
Epidemiology	7	136 (99.0-156.0)	7 (1.0-25.0)	2013 (2012.0-2016.0)	9 (6.0-10.0)	15 (12.2-17.3)
Pathogenesis	5	99 (97.0-193.5)	3 (0.0-6.0)	2011 (2010.5-2013.5)	11 (8.5-11.5)	10.2 (9.3-18.4)
Diagnosis - prognosis	2	124.5 (NA)	6.5 (NA)	2014.5 (NA)	7.5 (NA)	16.7 (NA)
Treatment	24	141 (111.25-197.75)	3 (1.0-12.5)	2011.5 (2010.0-2013.75)	10.5 (8.25-12.0)	13.6 (10.8-30.6)
Advanced imaging	5	94 (92.0-143.5)	1 (0.5-1.0)	2012 (2011.5-2014.5)	10 (7.5-10.5)	13 (9.8-14.8)
Clinic presentation	3	103 (NA)	221 (NA)	2015 (NA)	7 (NA)	17.1 (NA)

The journal with the most articles in the top 50 was "Ophthalmology", with 10 articles. The list of journals in which the top 50 uveitis articles were published and their detailed metrics are presented in Table 5 in the appendices. Journals in the ophthalmology category were the most published category with 29 articles and the median citation of WoS and median AAS were 126 (104.5-161.25) and 2 (0.25-7.75), respectively. Journals in the general medicine category had the highest median citations of 255.5 (154.0-279.25) and an AAS median of 209 (29.0-393.0). Elsevier was the publisher with the most articles, with 19 articles. The metrics by category of the journals in which the first 50 uveitis articles were published are summarized in Table 2.

**Table 2:** Journal metrics and changes by Q category of WoS and metrics by journal category Median (25%-75% interquartile range), NA: not applicable

Journal Category	N	Times cited of WoS	Altmetric Attention Score	Journal citation indicator	Impact factor	h-index	Normalized Eigenfactor score	Immediacy index	Article influence score
All Journals	50	123 (103.0-166.75)	3 (1.0-9.25)	2.64 (1.11-4.01)	7.389 (4.256-12.079)	196 (120.0-244.0)	5.327 (2.585-8.817)	4.126 (1.577-5.798)	2.781 (1.297-3.478)
Q1 category	36	130 (103.0-183.75)	4.5 (1.0-18.5)	3.91 (2.04-4.01)	12.079 (7.389-17.671)	242 (186.0-244.0)	8.769 (4.493-10.112)	5.798 (3.942-6.825)	3.478 (2.263-5.935)
Q2 category	9	112 (100.0-137.0)	1 (0.0-2.0)	1.02 (0.74-1.18)	4.123 (3.093-4.730)	110 (56.0-170.0)	2.826 (0.945-4.362)	1.434 (0.825-1.867)	1.042 (0.678-1.432)
Q3 category	4	117.5 (102.5-135.5)	3 (0.75-63.75)	0.79 (0.53-0.84)	2.602 (2.484-3.628)	75 (53.75-116.5)	0.692 (0.461-1.601)	0.638 (0.609-5.776)	0.696 (0.588-1.010)
Q4 category	1	139	3	0.60	2.454	41	1.531	0.609	0.682
Rheumatology	8	111 (98.0-136.75)	2 (1.0-5.25)	1.40 (1.12-2.70)	6.368 (4.698-10.485)	175.5 (153.25-295.5)	5.431 (3.639-8.961)	2.690 (1.202-4.487)	1.881 (1.388-3.370)
General medicine	6	255.5 (154.0-279.25)	209 (29.0-393.0)	26.14 (15.98-26.14)	91.253 (63.194-91.253)	1030 (693.5-1030.0)	132.478 (75.947-132.478)	162.030 (123.589-186.286)	37.313 (23.311-37.313)
Immunology	4	105.5 (98.25-121.0)	0.5 (0.0-3.25)	1.15 (0.63-1.65)	5.783 (4.095-14.288)	118.5 (69.5-200.5)	2.220 (1.353-4.510)	6.310 (2.359-12.706)	1.532 (1.054-5.491)
Ophthalmology	28	126 (104.5-161.25)	2 (0.25-7.75)	2.64 (1.60-4.01)	7.389 (4.256-12.079)	196 (120.0-244.0)	4.493 (2.585-8.769)	3.942 (1.582-5.798)	2.781 (1.297-3.478)
Genetics and hereditary	1	136	7	0.92	4.123	110	2.826	0.771	1.327
Cell biology	1	670	34	4.05	17.999	216	21.770	4.311	8.503
Biochemistry and molecular biology	1	113	3	0.45	2.607	94	0.617	0.646	0.764
Multidisciplinary sciences	1	92	1	0.57	3.240	332	226.378	0.619	1.011

**Figure 3:**
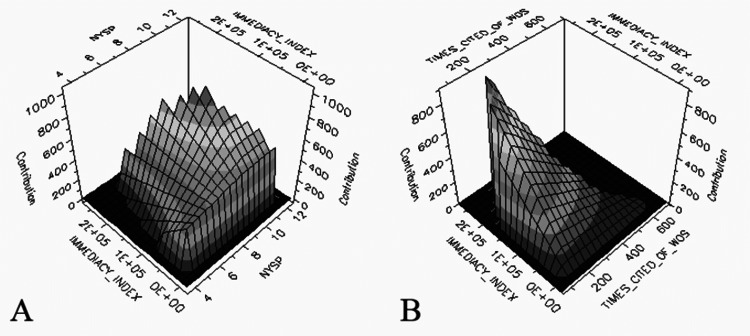
Three-dimensional representation of the contribution of the interaction of the predictors ((A) II and NYsP, (B) II and times cited in WoS) to the AAS in the MARS model II: Immediacy index; NYsP: number of years since publication; WoS: Web of Science; AAS: Altmetric Attention Score; MARS: multivariate adaptive regression splines

AAS was moderately correlated with IF, EF, AIS, JCI, and ACpY. AAS was weakly correlated with the number of citations and II. The correlation between AAS and article and journal metrics is summarized in Table 3. In the MARS model, the predictors are listed according to their importance scores calculated on the 100% scale, and the most important variable always receives a 100% score. Accordingly, the importance scores of the predictors are as follows: AIS (100%), II (77.74%), NYsP (57.79%), times cited in WoS (32.69%). In the model constructed with predictors, R2 was 0.97. The contribution of the interaction of the predictors to the model is presented in Figure 3.

**Table 3:** Correlation between AAS and article and journal metrics AAS: Altmetric Attention Score, WoS: Web of Science *significance level is p <0.05

Metrics	Spearman rho	p value
AAS – Times cited in WoS	0.326	0.021*
AAS – Publication year	0.285	0.045*
AAS – Average citations per year	0.487	<0.001*
AAS – Number of years since publication	-0.285	0.045*
AAS – Impact factor	0.476	<0.001*
AAS – 5-year impact factor	0.488	<0.001*
AAS – h-index	0.499	<0.001*
AAS – Journal citation indicator	0.436	0.002*
AAS – Normalized Eigenfactor score	0.466	0.001*
AAS – Article influence score	0.518	<0.001*
AAS – Immediacy index	0.346	0.014*

## Discussion

In this study, in which we evaluated articles related to uveitis, we found that the AAS was associated with citations, citation-based metrics, and the number of years since publication. The fact that AIS were the most important predictors indicates the importance of publishing an article in top journals. The fact that the II is the second-most important predictor reveals that the articles in the journals, which attract the attention of the scientific world faster, are more circulated in the online spaces. This was demonstrated by the fact that NYsP was the third-most important predictor in the regression model. However, caution should be exercised when interpreting these results. There was a time filter in the search strategy in this study. The NYsP might have been a more important predictor without the time filter since the provision of altmetric data began in 2011 [7]. Another thing to be aware of is the existence of social media accounts where journals share articles with their followers. In this way, they aim to make it possible for an article to reach a wider audience and attract attention [6, 8]. Finally, there may be predictors (e.g. popular topics) not included in the regression model.

The topic of most public interest may differ from that of the scientific community, and a more-cited article may not receive enough public attention [9]. Eight articles did not have high AAS in the altmetric analysis of glaucoma [10]. The fact that eight articles in the present study did not have high AAS is concrete proof that the agenda of the public and the scientific community is different. On the other hand, in the present study, it was observed that two case reports of uveitis caused by Zika virus and Ebola virus had a high AAS. The fact that case reports with a low level of scientific evidence compared to others have such a high AAS can be considered a result of the popularity of a disease affecting the public. In the study in which retinal articles in ophthalmology journals were evaluated, it was found that an article on retinal complications of Zika virus had the highest AAS [11]. In a study in which an altmetric analysis of 100 articles related to COVID-19 was performed, it was reported that the AAS was 3246 ± 3795 (85-16548) [12]. In a study evaluating the altmetric activity of 12.3 million WoS publications, it was reported that infectious diseases with a social impact received more attention [13]. AAS can also be interpreted as a social IF [14], and when this information is evaluated, we can say that topics that create social impact get more public attention than topics that are more technical. We can deduce that AAS may be low for a specific topic that concerns healthcare professionals. However, it should be kept in mind that as the popularity of the topic decreases, the speed of altmetric activity may slow down.

When the 10-year pool of articles included in the study is evaluated, it is seen that the majority of the articles are composed of NIUs, and treatment topics are predominant. In the past decade, the introduction of biological agents in the treatment of NIU has revolutionized the treatment. We noticed a concentration of treatment-related articles on adalimumab. Patients with uveitis are a relatively younger population, and uveitis has a more widespread effect on ocular morbidity than age-related macular degeneration [15]. Adalimumab is presented as a promising new treatment option with improvements in visual functionality outcomes [1, 16]. The article with the second-highest AAS (313) in the present study is associated with adalimumab. It may be due to patients wanting to know and understand their treatment, as well as wanting to explore new treatment options. On the other hand, journals in the general medicine category had the highest AAS. The fact that these non-ophthalmology journals are on our list and have a high AAS is proof that they are effective in both the ophthalmology community and the public.

The first limitation of our study was that we did not use a large data sample in the analysis. The second limitation was that we used a time filter. The third limitation was we used single-search terms. Despite these limitations, we have demonstrated the importance level of the effect of additional metrics on ASS.

## Conclusions

In conclusion, first, we found that the trend of articles on uveitis was related to the "treatment category"-adalimumab. Second, we found that the most popular topic was uveitis caused by viruses. Finally, we found that citation-based metrics and year of publication contributed to AAS. AAS appears to be inadequate in assessing the quality of articles. However, due to the electronic transformation of the publishing industry, it seems inevitable that altmetrics will become an additional supportive metric.

## Appendices

Tables 4-5 show the list of articles and journals included in this paper, respectively.

**Table 4:** Articles included in study

	Title	First Author	Years	Times cited	AAS
1	Effects of AIN457, a fully human antibody to interleukin-17a, on psoriasis, rheumatoid arthritis, and uveitis	Hueber, W	2010	670	34
2	Dexamethasone intravitreal implant for noninfectious intermediate or posterior uveitis	Lowder, C	2011	418	5
3	Adalimumab in patients with active noninfectious uveitis	Jaffe, GJ	2016	280	313
4	Persistence of Ebola virus in ocular fluid during convalescence	Varkey, JB	2015	279	633
5	A look at autoimmunity and inflammation in the eye	Caspi, RR	2010	274	8
6	Expert panel recommendations for the use of anti-tumor necrosis factor biologic agents in patients with ocular inflammatory disorders	Levy-Clarke, G	2014	267	8
7	Adalimumab for prevention of uveitic flare in patients with inactive non-infectious uveitis controlled by corticosteroids (VISUAL II): a multicentre, double-masked, randomised, placebo-controlled phase 3 trial	Nguyen, QD	2016	237	36
8	Secukinumab in the treatment of noninfectious uveitis: results of three randomized, controlled clinical trials	Dick, AD	2013	202	14
9	Randomized comparison of systemic anti-inflammatory therapy versus fluocinolone acetonide implant for intermediate, posterior, and panuveitis: the multicenter uveitis steroid treatment trial	Kempen, JH	2011	185	24
10	Understanding uveitis: the impact of research on visual outcomes	de Smet, MD	2011	180	0
11	Adalimumab plus methotrexate for uveitis in juvenile idiopathic arthritis	Ramanan, AV	2017	171	197
12	Interleukin-1 beta-regulating antibody XOMA 052 (gevokizumab) in the treatment of acute exacerbations of resistant uveitis of Behcet's disease: an open-label pilot study	Gul, A	2012	169	6
13	Cyclosporine for ocular inflammatory diseases	Kacmaz, RO	2010	166	3
14	Mycophenolate mofetil for ocular inflammation	Daniel, E	2010	162	0
15	A focus on the epidemiology of uveitis	Tsirouki, T	2018	159	0
16	Choroıdal evaluatıon using enhanced depth imagıng spectral-domain optical coherence tomography in Vogt-Koyanagi-Harada disease	Fong, AHC	2011	157	1
17	Incidence and prevalence of uveitis results from the pacific ocular inflammation study	Acharya, NR	2013	156	20
18	Treatment of refractory uveitis with adalimumab: a prospective multicenter study of 131 patients	Diaz-Llopis, M	2012	143	3
19	Review on the worldwide epidemiology of uveitis	Miserocchi, E	2013	140	84
20	Rituximab in intractable ocular lesions of behcet's disease; randomized single-blind control study (pilot study)	Davatchi, F	2010	139	3
21	Classification of intraocular tuberculosis	Gupta, A	2015	138	0
22	Uveitis- a rare disease often associated with systemic diseases and infections- a systematic review of 2619 patients	Barisani-Asenbauer, T	2012	136	7
23	Enhanced depth imagıng optıcal coherence tomography of the choroıd ın Vogt-Koyanagi-Harada disease	Nakayama, M	2012	130	0
24	Long-term remission after cessation of interferon-alpha treatment in patients with severe uveitis due to Behcet's disease	Deuter, CME	2010	130	1
25	Ocular signs predictive of tubercular uveitis	Gupta, A	2010	124	1
26	The 2009 prospective multi-center epidemiologic survey of uveitis in Japan	Ohguro, N	2012	122	3
27	Multicenter study of infliximab for refractory uveoretinitis in Behcet disease	Okada, AA	2012	119	1
28	The multicenter uveitis steroid treatment trial: rationale, design, and baseline characteristics	Kempen, JH	2010	115	3
29	Ocular toxoplasmosis past, present and new aspects of an old disease	Maenz, M	2014	113	1
30	Cytokines in autoimmune uveitis	Horai, R	2011	113	3
31	Prevention of flare recurrences in childhood-refractory chronic uveitis: an open-label comparative study of adalimumab versus infliximab	Simonini, G	2011	112	0
32	Abatacept for severe anti-tumor necrosis factor alpha refractory juvenile idiopathic arthritis-related uveitis	Zulian, F	2010	112	1
33	Long-term clinical outcome and causes of vision loss in patients with uveitis	Tomkins-Netzer, O	2014	111	13
34	Adalimumab successful in sarcoidosis patients with refractory chronic non-infectious uveitis	Erckens, RJ	2012	111	0
35	Cyclophosphamide for ocular inflammatory diseases	Pujari, SS	2010	109	0
36	Treatment of severe uveitis associated with juvenile idiopathic arthritis with anti-CD20 monoclonal antibody (rituximab)	Heiligenhaus, A	2011	108	1
37	Safety and efficacy of infliximab and adalimumab for refractory uveitis in juvenile idiopathic arthritis: 1-year followup data from the Italian registry	Zannin, ME	2013	104	3
38	Uveitis associated with Zika virus infection	Furtado, JM	2016	103	221
39	Risk of cataract development among children with juvenile idiopathic arthritis-related uveitis treated with topical corticosteroids	Thorne, JE	2010	103	1
40	Risk factors for loss of visual acuity among patients with uveitis associated with juvenile idiopathic arthritis: the systemic ımmunosuppressive therapy for eye diseases study	Gregory, AC	2013	99	1
41	Cytokine profiles in aqueous humor of patients with different clinical entities of endogenous uveitis	Abu El-Asrar, AM	2011	99	0
42	Immune mechanisms in inflammatory and degenerative eye disease	Perez, VL	2015	98	4
43	Serpiginous choroiditis and infectious multifocal serpiginoid choroiditis	Khanamiri, HN	2013	96	7
44	Inhibition of th17 differentiation by anti-tnf-alpha therapy in uveitis patients with Behcet's disease	Sugita, S	2012	96	1
45	Efficacy and safety of intravenous secukinumab in noninfectious uveitis requiring steroid-sparing ımmunosuppressive therapy	Letko, E	2015	94	5
46	Choroidal thickness in Behcet's uveitis: an enhanced depth imaging-optical coherence tomography and its association with angiographic changes	Kim, M	2013	94	1
47	Clinical and transcriptional response to the long-acting ınterleukin-1 blocker canakinumab in blau syndrome-related uveitis	Simonini, G	2013	94	6
48	Choroidal vascularity index (cvi) - a novel optical coherence tomography parameter for monitoring patients with panuveitis?	Agrawal, R	2016	92	1
49	Acute syphılıtıc posterior placoid chorioretinitis report of a case series and comprehensive review of the literature	Eandi, CM	2012	92	1
50	Prevalence of noninfectious uveitis in the United States a claims-based analysis	Thorne, JE	2016	90	25

**Table 5:** Journals evaluated in the study

Journal Name	Number of Articles	Impact Factor	H Index	Q Category
Ophthalmology	10	12.079	244	1
New England Journal of Medicine	4	91.253	1.030	1
Jama Ophthalmology (Archives of Ophthalmology)	4	7.389	196	1
American Journal of Ophthalmology	3	5.258	186	1
Retina-The Journal of Retinal and Vitreous Diseases	3	4.256	120	1
Progress in Retinal and Eye Research	2	21.198	152	1
Arthritis and Rheumatism	2	10.995	314	1
Arthritis Care & Research	2	4.794	163	2
Ocular Immunology and Inflammation	2	3.070	56	2
Lancet	1	79.323	762	1
Annals of the Rheumatic Diseases	1	19.103	240	1
Science Translational Medicine	1	17.992	216	1
Trends in Immunology	1	16.678	226	1
Journal of Clinical Investigation	1	14.808	488	1
Rheumatology	1	7.580	173	1
Survey of Ophthalmology	1	6.048	132	1
Arthritis Research & Therapy	1	5.156	150	2
Investigative Ophthalmology & Visual Science	1	4.799	218	1
Journal of Rheumatology	1	4.666	178	2
Orphanet Journal of Rare Diseases	1	4.123	110	2
Clinical Immunology	1	3.969	124	3
PLOS One	1	3.240	332	2
Graefes Archive for Clinical and Experimental Ophthalmology	1	3.117	101	2
Journal of Interferon and Cytokine Research	1	2.607	94	3
European Journal of Ophthalmology	1	2.597	53	3
International Journal of Rheumatic Diseases	1	2.454	41	4
Japanese Journal of Ophthalmology	1	2.447	56	3
